# Partial Ceramic Veneer Technique for Challenging Esthetic Frontal Restorative Procedures

**DOI:** 10.3390/dj11040101

**Published:** 2023-04-11

**Authors:** Gustavo Marotto Caetano, Cilea Slomp, Jonas Pereira Andrade, Ana Maria Spohr, Marcel Ferreira Kunrath

**Affiliations:** 1 Postgraduate Program in Dentistry, School of Health and Life Sciences, Pontifical Catholic University of Rio Grande do Sul (PUCRS), Porto Alegre 90619-900, Brazil; 2 Department of Biomaterials, Institute of Clinical Sciences, Sahlgrenska Academy, University of Gothenburg, 40530 Gothenburg, Sweden

**Keywords:** cervical veneer, partial veneer, esthetic rehabilitation, conservative ceramic, minimally invasive rehabilitation

## Abstract

Frontal darkened teeth have shown to be one of the most challenging treatments for esthetic dentistry in recent years. This case report, along with a 30-month follow-up, describes the application of a partial ceramic veneer, restricted to the mid-cervical third region, made in the upper left central incisor darkened by trauma. The procedure consisted of maintenance of the entire incisal face, as well as esthetic and morphological rehabilitation of the smile line with veneers and ultra-thin partial ceramic veneers. The planning process was thoroughly elaborated by photographs, study models, a virtual smile designed, diagnostic waxing, and reproduction in the patient’s mouth through mock-up. Following, dental home bleaching, esthetic gingival contouring, and minimally invasive tooth preparation were performed. Two types of glass ceramics (lithium disilicate and feldspathic) were used in different regions due to the peculiar characteristics of resistance, finishing, and polishing. According to the present case report, preparing a cervical partial ceramic veneer is an innovative, viable, and safe approach to maintaining the incisal portion of the tooth preserved and setting a detailed tooth morphology/color. The application of partial ceramic veneer provides incisal edge preservation and simplifies the reproduction of the adjacent tooth characteristics, avoiding multiple esthetic appointments.

## 1. Introduction

Dental trauma normally affects children and adolescents, with a prevalence of 4% to 33% of the world population [1,2]. The most common problems are pulp, periodontal, and esthetic complications [3,4], and the greatest occurrence is in the anterior teeth [5,6]. An esthetic intervention is considered necessary when there is a color change in the tooth, especially when there is pulpal sclerosis due to dental trauma [7].

External bleaching is one intervention technique that can be performed [8,9]. However, due to the unpredictability of this technique in determining the final color to be obtained and its recurrence over the years [10,11] and unsatisfactory outcomes reported by picky patients, other rehabilitation modalities have been proposed. Among them, ceramic crowns, which require preparation of the whole coronary portion and the consequent wear of the sound dental remnant, are traditionally used [12,13]. Nonetheless, the evolution of adhesive techniques and materials allowing greater tooth preservation through the preparation of ceramic veneers is being used [14,15,16]. As they allow only the buccal face of the affected tooth to be covered, they provide a favorable cosmetic recovery with highly proven clinical performance, according to several studies [17,18,19].

To maximize dental preservation with an esthetic and functional purpose, ultra-thin ceramic veneers ranging from 0.3 to 0.5 mm have recently been proposed [20,21,22]. Applying these veneers on whole dental faces has evolved to the production of partial ceramic veneers [23]. This modality requires greater precision and technical criteria, especially in relation to the final stages of finishing and polishing, to mask the interface between the ceramic and the enamel [24,25]. Hence, a zero-degree angular termination is desirable [26], differentiating the use of the partial over the total veneers. However, these techniques lack scientific studies and case follow-ups in the literature.

There is a great restorative challenge in cases of single anterior teeth with darkening on the cervical face only, which present anatomical and optical integrity of the incisal border. Ceramic stratification attempting to reproduce the same face of the homologous tooth precisely often produces unsatisfactory esthetic results [27,28]. It is also desirable to keep intact the dental structure present in the incisal region to preserve its richness of details [29].

Beyond the difficulty of mimicking the incisal region with ceramics and aiming for a minimally invasive approach, a restorative solution for substrates partially darkened by trauma can be the manufacture of partial ceramic veneers covering the affected zone.

## 2. Case Presentation

**Figure 1:**
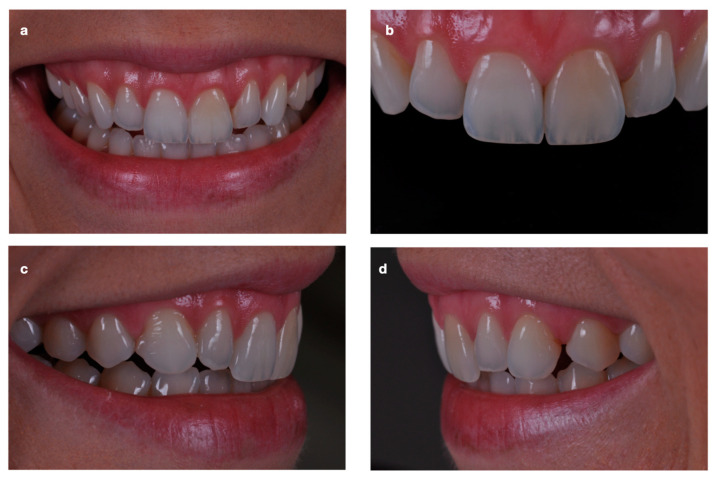
(**a**) Facial preoperative view of the smile. (**b**) Intraoral view revealing the darkening of the upper left central incisor tooth. The incisal aspect of the upper left central incisor is intact, with optic and morphological properties homologous to the upper right central incisor. (**c**) Lateral view showing composite resin restoration to close a diastema between the upper right canine and first upper right premolar. (**d**) Lateral view showing diastema between the upper left canine and first upper left premolar.

A 31-year-old female patient attended a private clinic with an esthetic complaint regarding the darkening of the upper left central incisor tooth (Figure 1a,b). This case report was evaluated and approved by the ethics committee of the Pontifical Catholic University of Rio Grande do Sul (PUCRS) (protocol number: 2.865.995). Additionally, the patient signed an informed consent agreeing with the report and exposition of your clinical case.

**Figure 2:**
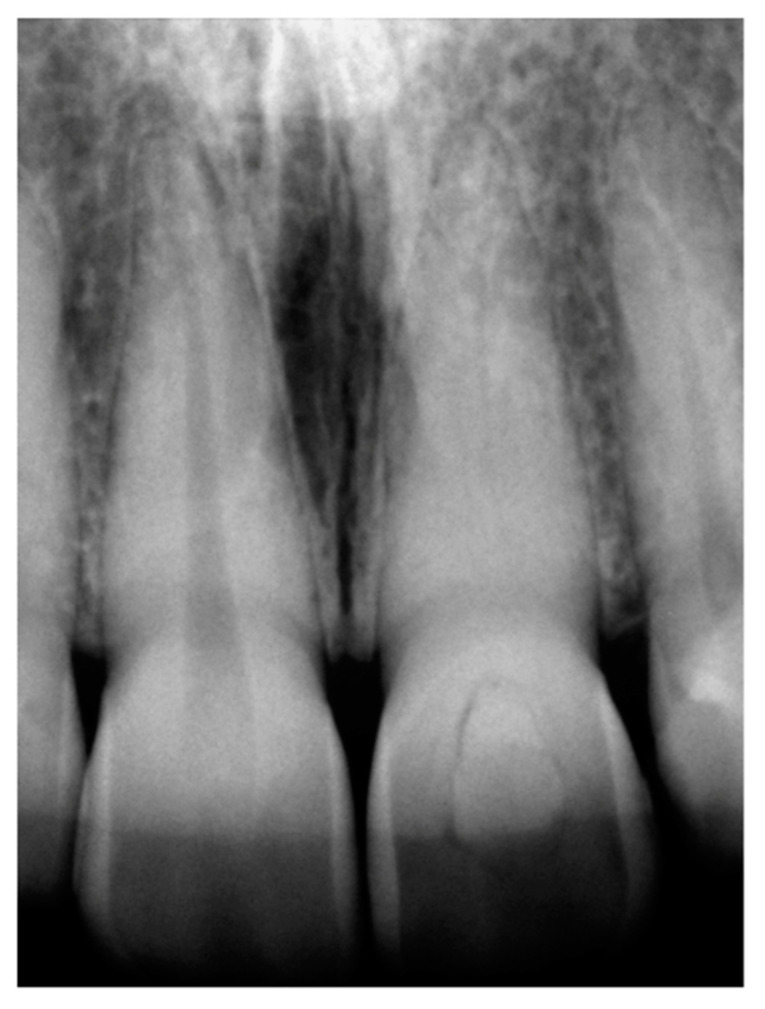
Periapical radiography showing obliteration of the pulp chamber and root canal of the upper left central incisor. Pulp sensitivity test showed positive reaction.

In the anamnesis, a previous incident of dental trauma in her adolescence was reported. The patient also reported having previously undergone, with a specialist, unsuccessful attempts (two) at bleaching and endodontics because of obliteration of the pulp chamber and root canal (Figure 2).

During clinical examination, it was observed that the upper left central incisor tooth had been restored with a composite resin on the lingual side, showing a darkening of the mid-cervical face. Its incisal aspect was intact, with optic and morphological properties homologous to the upper right central incisor, in which the incisal border was within the ideal esthetic parameters (Figure 1b). A pulp sensitivity test was carried out on the affected tooth, which responded positively. Radiographic examination revealed no periapical alteration (Figure 2).

The other teeth were healthy, with some having restorations made of a composite resin to close a diastema between the upper right canine and first upper right premolar (Figure 1c) and a diastema between the upper left canine and first upper left premolar (Figure 1d). Clinically, gingival esthetics was also compromised, causing disharmony in relation to the upper lip. The functional anterior and lateral dislocation guides were in agreement, with a mutual balanced occlusion.

**Figure 3:**
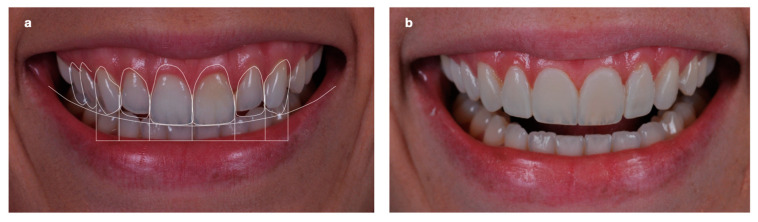
(**a**) Esthetic planning by a virtual analysis using DSD (Digital Smile Design). (**b**) Esthetic and functional evaluation by mock-up with bisacrylic resin.

The esthetic planning began by taking photographs of the face and intraoral (Figure 3), followed by a virtual analysis using DSD (Digital Smile Design) [30]. An impression with a vinyl polyxiloxane (Virtual, Ivoclar Vivadent, Schaan, Liechtenstein) was performed to obtain gypsum models. Subsequently, assembly of the models was carried out in a semi-adjustable articulator (Bio-Art, São Paulo, Brazil). Dental and gingival diagnoses were performed on the models according to the previous planning performed. Based on a guide made of vinyl polyxiloxane, diagnostic waxing was reproduced directly in the patient’s mouth. Esthetic and functional evaluation was obtained at the end by mock-up with bisacrylic resin (Protemp, 3M Espe, St. Paul, MN, USA). The patient approved this evaluation prior to the execution of the other rehabilitation procedures.

**Figure 4:**
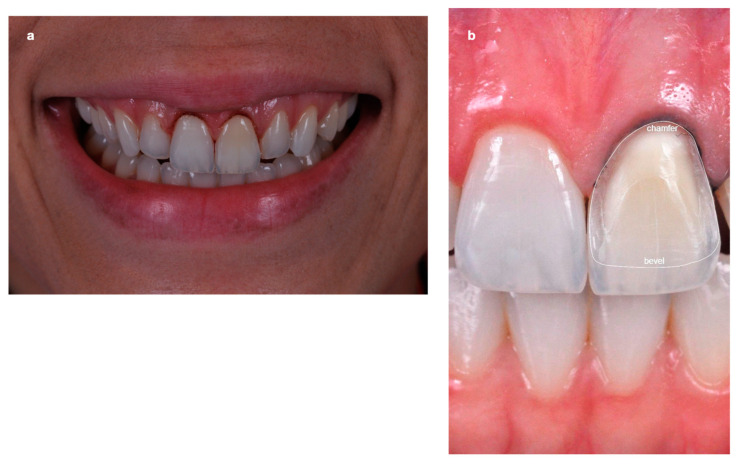
(**a**) Gingivoplasty performed for esthetic correction of the gingival contour of the upper right central incisor and upper left central incisor. (**b**) Preparation of the upper left central incisor. Greater wear in the cervical third was performed, being finished at zero degrees in the margins and towards the incisal third.

Initially, the treatment consisted of daily home bleaching with 16% carbamide peroxide (FGM, Joinville, SC, Brazil) for three weeks. Flapless gingivoplasty was then performed for the esthetic correction of the gingival contour of the upper right central incisor and upper left central incisor (Figure 4a) without bone removal.

After three weeks of gingival healing, dental preparations for the ceramic veneers were made in the anterior teeth of the maxilla and premolars, except for the upper right central incisor and second upper left premolar. The upper right central incisor remained intact and did not receive any treatment because it did not show an esthetic compromise. Preparation of the upper left central incisor consisted of greater wear in the cervical third using burs 2135/2135F, 1.0 mm on average, being finished at zero degree in the margins and towards the incisal third, aiming preservation of the healthy border without wear due to its similarity to the homologous tooth (Figure 4b).

**Figure 5:**
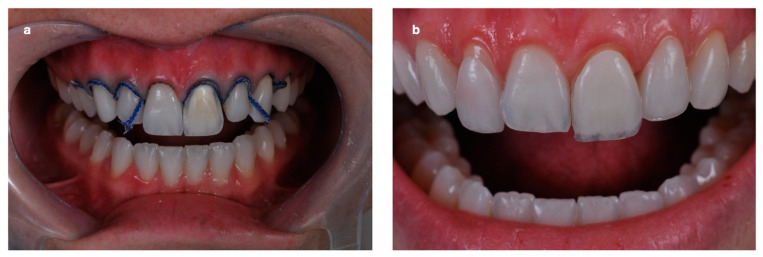
(**a**) Intra-sulcular cords positioned before impression with vinyl polyxiloxane. (**b**) Feldspathic ceramic veneer being positioned for luting procedure on the upper left incisor. Visible transparency on the incisal region can be noticed due to the methodology applied in this case promoting a ceramic finishing line at zero-degree.

Afterward, the impression was performed with intra-sulcular cords (Ultradent, South Ultradent, UT, USA), intra-sulcular #000, and overlaid #1 (Figure 5a), followed by a vinyl polyxiloxane application (Virtual, Ivoclar Vivadent, Schaan, Liechtenstein). Subsequently, the provisional restorations were obtained using bisacrylic resin (Protemp A1, 3M ESPE, St. Paul, MN, USA) on the previous silicone guide.

The ceramic restorations were made at a dental prosthesis laboratory and varied according to the technique used. Ceramic veneers were made for the buccal surfaces of the upper right canine, first and second upper right premolars, and upper left central incisor. Moreover, a partial ceramic veneer was obtained for the distal face of the upper left canine and the mesial face of the first upper left premolar. Feldspathic ceramic was chosen (Noritake EX3, Suita, Osaka, Japan) for these teeth. A veneer covering the entire buccal face was made for the upper left central incisor, with a greater thickness (1.0 mm) in the most chromatic area of the preparation, an intermediate thickness on the middle third (0.5 mm) and is finished in zero-degree at the incisal border.

For the upper left and right lateral incisors, lithium disilicate ceramic veneers were made in an HTBL3 color (e.Max Press, IvoclarVivadent, Schaan, Liechtenstein). Afterward, they received a thin layer of stratification on the buccal face with feldspathic ceramic (Noritake EX3) (Figure 5b).

**Figure 6:**
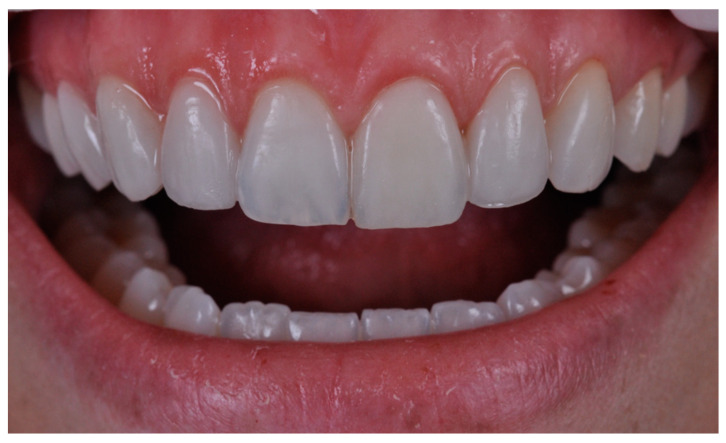
Immediate photo after luting and polishing procedures in frontal smile.

The ceramic restorations were luted with Variolink Esthetic (IvoclarVivadent, Schaan, Liechtenstein), neutral shade, following the adhesive luting protocol recommended by the manufacturer. The partial veneers were then finished and polished directly in the mouth using 3118 FF, 3216 FF diamond burs (KG Sorensen, Cotia, SP, Brazil) and Eve silicon abrasives—disk format (Neureutstr, Keltern, Germany) in the following order: blue, pink, and gray using a rotation speed of 1200 rpm; moreover, a magnifier (2.5 × zoom, ExamVision, Copenhagen, Denmark) was applied for careful detailing. The ceramic veneer of the upper left central incisor was transformed into a partial ceramic veneer by intraoral wear in the incisal third and partially in the middle third, finishing at zero degrees in the incisal direction (Figure 6). After this step, the transition between tooth and ceramic was visually imperceptible.

The protrusive and laterotrusive functional guides were preserved and adjusted, resulting in a mutually protected occlusion at the end of the treatment. After the treatment, the patient was instructed regarding oral hygiene and periodic visits every six months, apart from periodic polishing of the partial veneer when considered necessary (recommended annual visit for ceramic polishing).

**Figure 7:**
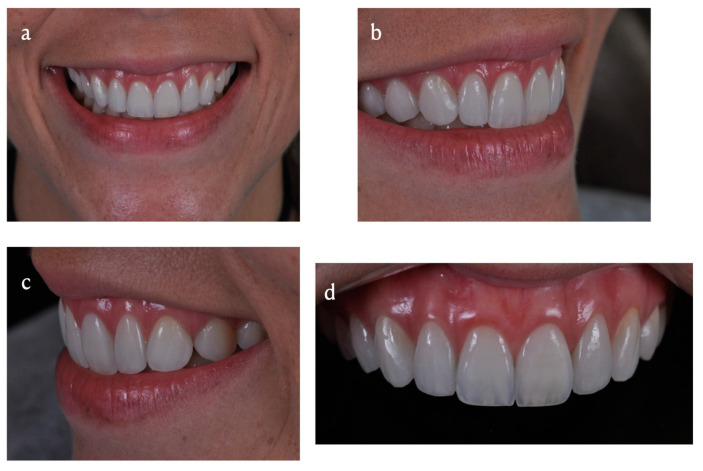
Postoperative views after 30 months, showing maintenance of the ideal biological, functional, and esthetic conditions. (**a**) Extraoral frontal smile. (**b**,**c**) Lateral extraoral views. (**d**) Intra-oral frontal photo showing maintenance of esthetic conditions after re-polishing.

Follow-up was performed 30 months after the conclusion of the treatment, which clinically demonstrated maintenance of the ideal biological, functional, and esthetic conditions of the successful rehabilitation treatment (Figure 7).

## 3. Discussion

Masking the remaining darkened teeth is a hard task for the clinician and laboratory technician. The etiology of this darkening may help predict the degree of tissue response to the proposed interventions, such as bleaching procedures [31,32]. However, the degree to which this tooth will be exactly whitened at the end of the process cannot be defined [33,34]. In addition, recurrence of the initial color may present over the years, or even little prediction of the results [33], requiring frequent repairs required by picky patients. This fact contributed to choosing a definitive basis treatment in the case proposed, with the restoration in ceramic for masking the darkened substrate. However, non-invasive treatments should be prioritized before any definitive treatment aiming integrity of teeth; after unsuccessful outcomes, definitive treatments should be suggested with the patient’s agreement. 

Dental trauma is one of the main causes of dental darkening [35]. Along with that, the presence of pulp vitality is more frequently found than necrosis [6,36]. In the present case, an endodontic treatment was attempted because the dental pulp was undergoing a sclerosis process. However, there was a painful sensation at the moment of the coronary opening, and the root canal obliteration was verified. Because of this, the endodontic procedure was not carried out, but only clinical and radiographic control at the end of the esthetic rehabilitation. Following the precepts of conservative dentistry, the integrity of the root canal after trauma was prioritized in agreement with the literature [6].

The new approach and evolution of adhesive dentistry and new dental preparation philosophies for enamel have caused a growing demand for highly conservative esthetic treatments, obtaining maximum structural preservation. Considering this, the thickness of the ceramic veneers can be reduced from the one commonly reported in dental veneers (1–1.5 mm) to 0.3 mm [37]. At the same time, the application of these veneers can be limited only to the specific desirable tooth area by partially covering its structure, thereby configuring the so-called partial veneer [26]. Although its most cited indication is for morphological adaptations [38], such as the closure of diastemas described in the present case, the use of partial veneers involving only the cervical face of teeth has also been reported [37]. However, such a study refers to the purpose of covering non-carious lesions rather than changing the final tooth color obtained in esthetic treatments, which was proposed in this case.

The choice of the ceramic type should be made based on some criteria, among them the mechanical and esthetic properties and the processing method [39]. One complexity presented in the skills of the case proposed was making the veneers with two ceramic systems, which were separately implied and executed in different techniques. The systems were represented by the feldspathic ceramic, which was manually stratified, and the injected lithium disilicate ceramic, later characterized with pigments in the refractory die.

The lithium disilicate ceramic veneer technique was chosen for the upper left and right lateral incisors because of its higher intrinsic mechanical resistance [40]. It is desirable because both teeth, in this case, received an increase in their incisal heights. In the case of the darkened upper left central incisor, feldspathic ceramics were used due to the possibility of stratification in increments with different opacities and color saturation in successive layers, simulating enamel and dentin [41]. This provided a better reproduction of the characteristics of the homologous tooth through the artistically developed sculpture. Another purpose for this tooth was to allow adequate finishing and polishing at the ceramic–resin cement–enamel interface, a fundamental requirement in this partial veneer. Because it contains more glass and fewer crystals, feldspathic ceramic facilitates the finishing steps in the mouth of the beveled end of the partial ceramic veneer [41,42].

When there is an esthetic impairment on a single central incisor tooth, the use of ceramic restorations is normally indicated in both central incisors to obtain the best esthetic result [43]. However, aiming at a minimally invasive ideology, in the case described, a single restoration was performed on the upper left central incisor, maintaining the integrity of the upper right central incisor, as it had rich esthetic details that deserved to be reproduced in its darkened counterpart. The incisal border may be considered a prime region of a tooth that has not received attrition over the years, and as such, dentists seek to maintain it intact [29,43]. Studies recommend an incisal reduction of around 1.5 to 2 mm as a way of obtaining greater mechanical resistance and reproduction of the anatomical details in teeth with veneers [29]. However, the upper left central incisor revealed an incisal edge with desirable translucency, opalescence, anatomical texture, and color, and there was no need for ceramic coating. Therefore, the preparation of the border was contraindicated. Consequently, it was chosen the preservation of the tooth and the adaptation of a partial veneer involving only its mid-cervical face instead of a total buccal coverage veneer.

As for the case, thin and ultra-thin veneers (0.3–0.7 mm) could not mask the color of the darkened substrates [14,20,28,42]. Then, the dental preparation consisted of slightly greater wear in the more saturated region in the cervical face of the tooth.

A veneer was initially made to acquire the final configuration of a single cervical partial veneer covering the entire buccal surface. It was subsequently abraded, finished, and polished in the incisal and middle thirds directly in the mouth after luting. Finally, it was transformed into a partial veneer. The partial ceramic veneer consisted of a greater thickness in the more chromatic area of the preparation and obtained a level termination in the middle of the incisal third. The purpose of finishing and polishing with the intraoral technique was to ensure a marginal termination at a zero-degree angle and promote the absence of a visual luting line. This approach is efficient and innovative, but unfortunately, there are no long-term studies up to now, and it is not a habitual clinical routine such as procedures using composite resins, showing a limitation for this specific approach [44]. Currently, studies describe total darkened teeth treated with total veneer and not with partial veneer, as in the present case report [45].

The success of the treatment conducted was possible due to adequate planning. Maintenance of the esthetic and functional requirements after 30 months and a longitudinal follow-up of the present case proved successful. Additionally, the cooperation between patient and dentist maintaining annual appointments for re-polishing ceramics and marginal termination is of paramount importance.

## 4. Conclusions

Despite the limitations of this single case report, it can be concluded that preparing a cervical partial ceramic veneer is an innovative, viable, and safe approach after a short-term follow-up.

Additionally, this technique demonstrated a minimally invasive approach to preserve the incisal edge of the tooth, maintaining the natural characteristics and simplifying the laboratory technician work, reducing the possible number of re-appointments in order to achieve the correct tooth color and tooth morphology. However, minimally invasive esthetic rehabilitation with conservative ceramic veneers requires adequate planning and the appropriate skill for its success. Furthermore, long-term follow-ups and multiple cases should be investigated in order to confirm the findings revealed in this report. Moreover, non-invasive treatments such as bleaching protocols should be prioritized before any tooth wear, evolving to prosthetic treatments after unsuccessful results if agreed with patients. 

## Data Availability

All data created with this study is reported and shared in this article.
